# Association of ambient temperature with intentional self-harm and suicide death in Seoul: a case-crossover design with a distributed lag nonlinear model

**DOI:** 10.1007/s00484-024-02752-z

**Published:** 2024-08-23

**Authors:** Seunghyeon Kim, Yoonhee Kim, Eunsik Park

**Affiliations:** 1 Department of Mathematics and Statistics, Chonnam National University, 77 Yongbong-ro, Buk-gu, Gwangju, 61186 Korea; 2 Department of Global Environmental Health, Graduate School of Medicine, The University of Tokyo, Tokyo, 113-0033 Japan

**Keywords:** Intentional self-harm, Suicide death, Ambient mean temperature, Meteorological factors, Case-crossover design, Distributed lag nonlinear model

## Abstract

Previous epidemiological studies have reported a short-term association between ambient temperature and suicide risk. To gain a clearer understanding of this association, it is essential to differentiate the risk factors for intentional self-harm (ISH) from those specifically associated with suicide deaths. Therefore, this study aims to examine whether the association between daily temperature and ISH or suicide deaths differs by age and sex. Between 2014 and 2019, cases of emergency room visits related to ISH and suicide deaths in Seoul were identified. A time-stratified case-crossover design was used to adjust for temporal trends and seasonal variation. A distributed lag nonlinear model was used to analyze the nonlinear and time-delayed effect of ambient temperature on ISH and suicide deaths. Positive associations were observed between temperature and both ISH and suicide deaths. For ISH, the relative risk (RR) was high at 1.17 (95% confidence interval (CI): 1.03, 1.34) for a temperature of 25.7 °C compared with 14.8 °C. The RR for suicide death was higher than those for ISH, at 1.43 (95% CI: 1.03, 2.00) for a temperature of 33.7 °C. These associations varied by age and sex, with males and females aged 35–64 years showing increased susceptibility to suicide deaths. This study provides detailed evidence that unusually high temperatures, both anomalous and out of season, may trigger suicidal behaviors, including both ISH and suicide deaths.

**Supplementary Information:**

The online version contains supplementary material available at 10.1007/s00484-024-02752-z.

## Introduction

Climate change is increasingly recognized as a critical societal issue. The 2021 IPCC Sixth Assessment Report provides clear evidence of the ongoing influence of climate change (Arias et al. 2021). In 2020, global average temperatures were 1.2 degrees Celsius higher the of 1850–1900 baseline, with the years 2015 to 2020 being the six warmest on record (Caretta et al. 2022). South Korea (hereafter, Korea) experienced an even more pronounced temperature increase, rising approximately 1.8 degrees from 1912 to 2017 (KMA, 2020). This represents a 29% higher rate of increase than the global average temperature rise of approximately 1.4 degrees over the same period.

The relationship between climate change and suicide requires serious attention. Climate change influences mental health directly and indirectly (Caretta et al. 2022). High temperatures have been linked to an increase in mental health-related emergency room visits (Mullins and White 2019; Wang et al. 2014), and incidents of self-harm (Akkaya-Kalayci et al. 2017; Kubo et al. 2021; Lee et al. 2020; Williams et al. 2016), including suicides (Belova et al. 2022; Carleton 2017; Casas et al. 2022; Frangione et al. 2022; Gao et al. 2019; Kalkstein et al. 2019; Kim et al. 2011, 2016, 2019; Schneider et al. 2020). A study conducted among U.S. populations revealed that a temperature increases of 1–6 °C could result in an annual increase in suicides ranging from 283 to 1,660 (Belova et al. 2022). These findings highlight the importance of recognizing suicide as a significant mental health concern, that climatic changes may influence.

Suicide is a significant and persistent societal challenge. Globally, over 700,000 people die by suicide each year (WHO, 2023). This issue is especially pressing in Korea, where the suicide rate was 25.4 per 100,000 people in 2019—the highest among the Organization for Economic Co-operation and Development (OECD) countries (OECD, 2023). In Korea, the rate of suicide-related deaths increased by 46% between 2000 and 2019, in stark contrast, to the 29% decline observed across the OECD nations during the same period (OECD, 2021).

The alarming suicide rate in Korea is influenced by several interconnected factors contribute to, such as intense academic pressure on youth, inadequate social services and support for the elderly, widening income disparities, and declining social cohesion (Kwak and Ickovics 2019; Kwon et al. 2009). Further, meteorological elements, including ambient temperature and relative humidity, have been investigated as potential triggers for suicidal behaviors. While the connection is uncertain, the widespread presence of these factors could make them potentially risky (Frangione et al. 2022).

Intentional self-harm (ISH) and suicide deaths are distinct events, each with unique characteristics. Self-harm often involves an “episode of ISH that may or may not be driven by a desire to end life” (Bebbington et al. 2010). ISH is considered one of the most significant risk factors for future suicide (Hawton et al. 2003), underscoring the importance of examining factors related to ISH and suicide deaths.

The characteristics of ISH and suicide deaths also differ by sex. The “gender paradox of suicidal behavior” suggests that while women are more prone to ISH than men, they are more likely to survive such attempts (Mergl et al. 2015; Schrijvers et al. 2012). In Korea, despite more women than men engaging in ISH, the incidence of suicidal deaths is significantly higher among men, being twice as prevalent compared to women (Seong et al. 2022). Previous studies have explored whether socioeconomic risk factors differ between the suicide attempt and completed suicide, revealing varied effects across different sexes and age groups. These studies indicate that income levels significantly influence men, while lower levels of education emerged as a risk factor for suicide attempts and completed suicides, especially among women and younger individuals (Raschke et al. 2022). However, research investigating how meteorological factors influence suicide attempts and completed suicides across different sexes and age groups remains lacking.

Expanding on the preceding context, the following hypotheses were developed: (1) Short-term exposure to elevated temperatures may prompt visits to emergency room visits for ISH and increase suicides; (2) Temperature likely has a stronger association with severe outcomes, such as suicide death; and (3) these associations may vary across sexes and age groups.

Therefore, this observational epidemiology study aims to investigate emergency room visits resulting from ISH and suicide death cases in Seoul, Korea—a region characterized by the highest suicide rate among OECD nations (OECD, 2021). These findings enhance our understanding of the distinct characteristics of ISH and suicide deaths. They provide additional evidence to clarify the ambiguous relationship between ISH and temperature.

## Materials and methods

### Data source

The ISH data were sourced from the National Emergency Department Information System (NEDIS), a standardized data registration and transmission system established by the National Emergency Medical Center (NEMC) (https://www.e-gen.or.kr/english/main.do). Established in 2003 under Articles 15 and 17 of the Emergency Medical Service Act, NEDIS collects real-time data on medical treatments provided to individuals visiting emergency departments at designated medical institutions. This includes cases of illness, non-illness, and non-medical visits. The data collected by NEDIS supports research and informs policy formulation (NEMC, 2019). Emergency departments are categorized into three types: regional emergency medical centers (6.9%, average percent of total period), local emergency medical centers (53.1%), and local emergency medical agencies (40.0%). In Seoul, the participation rate in the NEDIS database was approximately 90.7% for 2014 and 2015, increasing to 100% from 2016 to 2019. Each emergency department contributed the following details to the NEMC database: (1) demographics details, including age, sex, and insurance types (such as health and car insurances); (2) emergency room specifics, including location and type of emergency medical institution; (3) medical data, including the date and time of the emergency room visit and the onset of the main symptom; and (4) suicide-related information, including intentionality (such as unintentional, intentional self-harm, suicide, violence, and assault), emergency treatment outcomes (such as discharge or death), mechanisms of injury (such as fall, poisoning, and firearm/cut[sharp objects]/pierce), and post-hospitalization results (such as normal discharge, voluntary discharge, and death).

Statistics Korea provided comprehensive data on suicide deaths in the country, including (1) Demographic Details: Such as age, sex, occupation, education level, and marital status of the deceased individuals. (2) Medical Data: Including the address of the deceased and date of death. and (3) Suicide-Specific Information: Such as the specific cause of death related to suicides.

Meteorological data, including daily mean, minimum, and maximum temperatures, along with mean dewpoint temperatures, were obtained from the Korea Meteorological Administration (https://data.kma.go.kr/cmmn/main.do). These data were collected from a single monitoring station at the Seoul Observatory, part of the Automated Synoptic Observing System, which performs ground-based observations to assess weather on a synoptic scale. Seoul, the capital of Korea, situated at 37.57° N latitude and 126.98° E longitude. According to the Köppen-Geiger classification, Seoul has a hot-summer humid continental climate. Summers in Seoul are hot and humid owing to the influence of the North Pacific high-pressure system with temperatures occasionally approaching 40 °C. Conversely, winters are cold influenced by the Siberian High and prevailing westerlies, with temperatures sometimes dropping to approximately − 20 °C during severe cold spells. Seoul, with an estimated population of 10 million and a density of approximately 16,000 individuals per square kilometer, provides a comprehensive setting for evaluating the association between ambient temperature and suicide owing to its significant population and temperature fluctuations.

### Outcomes of interest

**Fig. 1:**
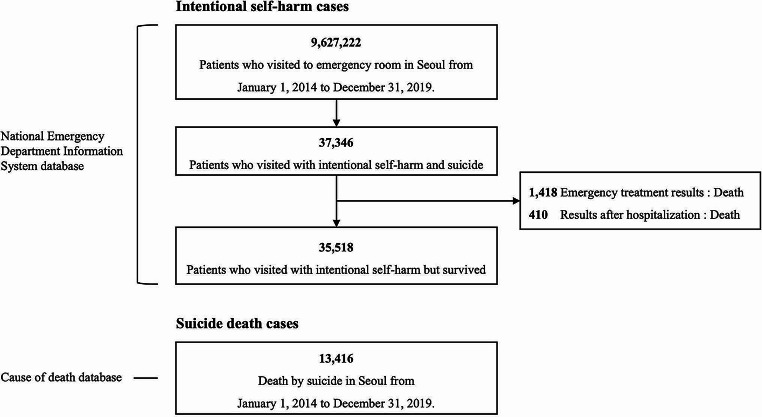
Flowchart depicting the selection of study population in Seoul (2014–2019). The study comprised 48,934 suicide cases, including 35,518 non-fatal ISH visits and 13,416 suicide deaths

This study examines emergency room visits associated with ISH and deaths by suicide in Seoul from 2014 to 2019. Emergency room visits for ISH are identified in the NEDIS database under the “intentionality” variable, specifically categorized as ISH and suicide. The category includes both individuals who survived and those who died from ISH. Additionally, individuals who died are recorded as deaths by suicide in the Statistics Korea database. Non-fatal cases and fatal cases were partitioned. For non-fatal cases, only ISH instances that did not result in suicide deaths were included. This was done by excluding death cases based on the “emergency treatment result” and “post-hospitalization result” variables. Figure 1 shows ISH (non-fatal cases) and suicide death (fatal-cases) by specific inclusion and exclusion criteria. All ISH cases, including both survivors and deceased, totaled 36,936, excluding 410 cases in the NEDIS dataset where the outcome was death in “post-hospitalization result” variables.

Suicide deaths in Seoul were identified as cases of ISH using the ICD-10 codes X60–X84, according to the Cause of Death database maintained by Statistics Korea.

### Study design

The time-stratified case-crossover design was used to study the short-term association between ambient temperature and ISH, along with suicide death. This self-matched design is a specialized version of the case-control design commonly used in environmental epidemiology. It assesses the short-term effects of environmental factors, including temperature, on the risk of acute diseases. By using each individual as their own control, the design adjusts for confounding factors that remain constant over time, such as age and sex (Maclure et al. 2000). The time-stratified case-crossover design is particularly effective in reducing biases from time trends, seasonal variations, and the non-independent selection of control days (Wu et al. 2021). In this study, each case day (i.e., the day of an ISH incident and suicide death) was matched with another day of the week within the same month and year.

### Statistical analysis

To examine the relationship between ambient temperature and both ISH and suicide deaths, a conditional quasi-Poisson regression model was used in conjunction with a distributed lag nonlinear model (DLNM) (Gasparrini 2011; Gasparrini et al. 2010). While estimates from conditional quasi-Poisson regression models are comparable to those from conditional logistic regression models, they also account for overdispersion (Armstrong et al. 2014). The model can be expressed using the following equation:

$${Y_t} \sim quasi - Poisson\left( {{u_t}} \right),$$

$$\eqalign{ Log\left( {{u_t}} \right) & = \alpha + \;\beta {T_{t,l}} + S\left( {D{T_t},3} \right) \cr & \quad + \lambda Stratu{m_t} + \eta Holida{y_t} \cr} $$

where $\:{Y}_{t}$ represents the expected number of ISH or suicide death occurrences on day $\:t$; $\:\alpha\:$ is the intercept; $\:{T}_{t,l}$ is a cross-basis matrix acquired by applying DLNM to the mean temperature; $\:\beta\:$ is the vector of coefficients for $\:{T}_{t,l}$; $\:l$ refers to the lag days; and $\:S\left(\:\right)$ represents a natural cubic spline. For suicide death cases, the basis function for the exposure dimension was a natural cubic spline with an internal knot at the 50th percentiles of mean temperature. For ISH, three internal knots located at the 25th, 50th, and 75th percentiles of mean temperature were used. These choices were guided by the Quasi Akaike Information Criterion (Supplementary Table S1; Gasparrini et al. 2010). In the parallax space, a natural cubic spline function was used with knots spaced evenly on a logarithmic scale, accommodating a total lag of 2 days. The lag-exposure plot shows that a maximum lag of 2 days effectively captures the lag effect (Supplementary Fig. S1). The variable $\:{Stratum}_{t}$ represents the day of the week within the same month and year. Using a time-stratified case-crossover design allows for inherent control over weekly patterns, seasonality, and long-term trends. $\:{DT}_{t}$ represents the daily mean dewpoint temperature, while $\:{Holiday}_{t}$ is a binary variable set to “1” if day $\:t$ is a holiday. Dewpoint temperature, the temperature at which air reaches 100% relative humidity, is considered a superior indicator of air humidity compared to relative humidity (Davis et al. 2016; Li et al. 2020).

To better estimate the relationship between temperature-ISH and temperature-suicide death, the maximum risk temperature (MaxRT) was identified within the 1st to 99th percentiles of the temperature distribution, indicating the highest risk for these outcomes. Relative risks (RRs) are presented in two ways: (1) the RR at MaxRT compared to the 50th percentile of mean termperature; (2) the RR at the 99th percentile of mean temperature compared to the 50th percentile. The latter method was used to compare RRs across subgroups. All RRs are reported with 95% confidence intervals.

The study population was divided into three age groups based on a previous Korean study: 0–34, 35–64, and ≥ 65 years old (Kalkstein et al. 2019). Various combinations of sex and age groups were then analyzed, with statistical analyses performed for each subgroups.

Additionally, a sensitivity analysis was conducted to assess the robustness of the results. This analysis involved changing the maximum lag period from 2 days to 3 or 6 days. It also included replacing mean temperature with minimum and maximum temperatures. All statistical models were fitted using R (version 4.0.3) software, with the “dlnm” and “gnm” packages.

## Results

### Descriptive analysis

Table 1 shows the count and proportion of ISH and suicide deaths by subgroup from 2014 to 2019 in Seoul. During this period, 35,518 cases of ISH and 13,416 cases of suicide deaths were documented. Among the ISH cases, females represented 59.4% of the total, and the highest occurrence rate was observed in the aged 35–64 years group, accounting for 47.5%. The group aged 0–34 years followed with 40.1%. Regarding methods of ISH, poisoning was the most common, comprising 52.9% of cases, while sharp objects were used in 27.6% of cases. Conversely, among suicide deaths, males represented a higher proportion at 68.7%. Individuals 35–64 years made up 54.7% of the total, while those aged ≥ 65 constituted 25.1%. Additionally, 39.7% of all suicide deaths occurred among men aged 35–64 years. The most common methods of suicide were hanging (53.0%), poisoning (19.2%), and jumping (18.5%). The average daily mean temperature in Seoul from 2014 to 2019 was 13.4 ± 10.7 °C, with an average daily mean dewpoint temperature of 4.7 ± 12.0 °C (Table 2).

**Table 1:** Descriptive statistics of daily intentional self-harm and suicide death cases in Seoul (2014–2019)

Variables	Intentional self-harm		Suicide deaths	
	Count (*n*)	Percentile (%)	Count (*n*)	Percentile (%)
*Total*	35,518	100.0	13,416	100.0
*Sex*				
Female	21,092	59.4	4,205	31.3
Male	14,426	40.6	9,211	68.7
*Age (years)*				
0–34	14,241	40.1	2,715	20.2
35–64	16,887	47.5	7,340	54.7
≥ 65	4,390	12.4	3,361	25.1
*Sex/Age*				
Female, 0–34	9,120	25.7	1,140	8.5
Female, 35–64	9,790	27.6	2,014	15.0
Female, ≥ 65	2,182	6.1	1,051	7.8
Male, 0–34	5,121	14.4	1,575	11.7
Male, 35–64	7,097	20.0	5,326	39.7
Male, ≥ 65	2,208	6.2	2,310	17.2
*Methods*				
Poisoning	18,775	52.9	2,577	19.2
Drowning	690	1.9	876	6.5
Hanging	1,358	3.8	7,117	53.0
Jumping	726	2.0	2,483	18.5
Sharp objects	9,791	27.6	151	1.1
Others	4,178	11.8	212	1.6

**Fig. 2:**
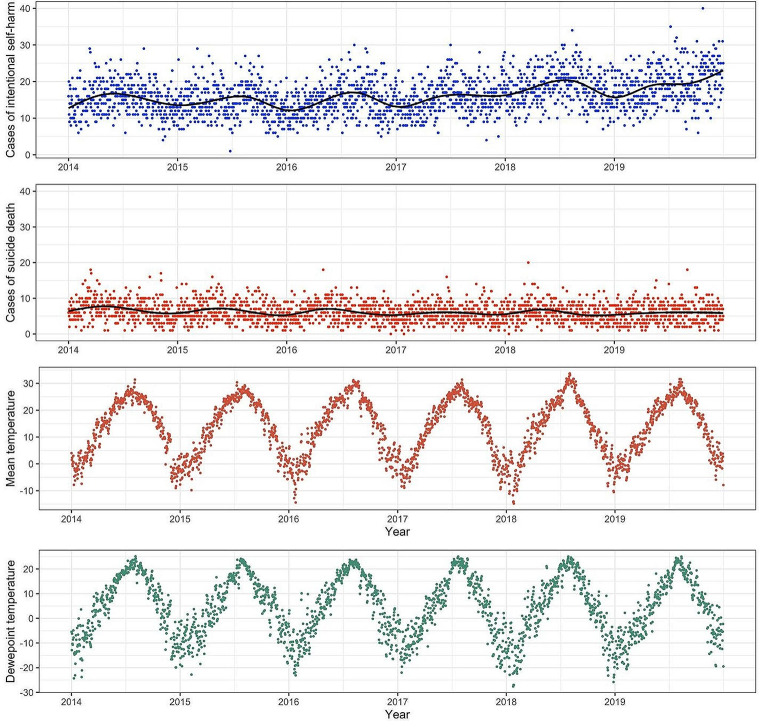
Time series plots of intentional self-harm cases, suicide death, and meteorological factors in Seoul. *Note: The black line in the top two figures represents the natural cubic spline function with degrees of freedom equal to 3 per year, which smoothly captures seasonal trends

Figure 2 display a daily time series plot from 2014 to 2019, showing ISH, suicide deaths, mean temperatures, and mean dewpoint temperature. A significant finding from the data reveals that ISH and suicide deaths occurred more frequently during periods of higher temperatures. While suicide deaths showed a consistent annual pattern, ISH cases noticeably increased starting from 2016.

**Table 2:** Daily statistical summary of meteorological factors in Seoul (2014–2019)

	Mean ± SD	Minimum	*P*_25_	Median	*P*_75_	Maximum
Mean temperature (°C)	13.4 ± 10.7	−14.8	4.0	14.8	22.9	33.7
Minimum temperature (°C)	9.2 ± 10.8	−18.0	−0.2	10.1	18.7	30.3
Maximum temperature (°C)	18.4 ± 10.9	−10.7	8.5	20.4	27.9	39.6
Dewpoint temperature (°C)	4.7 ± 12.0	-27.6	-5.1	5.8	15.1	25.1

SD, standard deviation; P_x_, x^th^ percentile

### Distributed lagged effects of temperature on intentional self-harm and suicide deaths

**Fig. 3:**
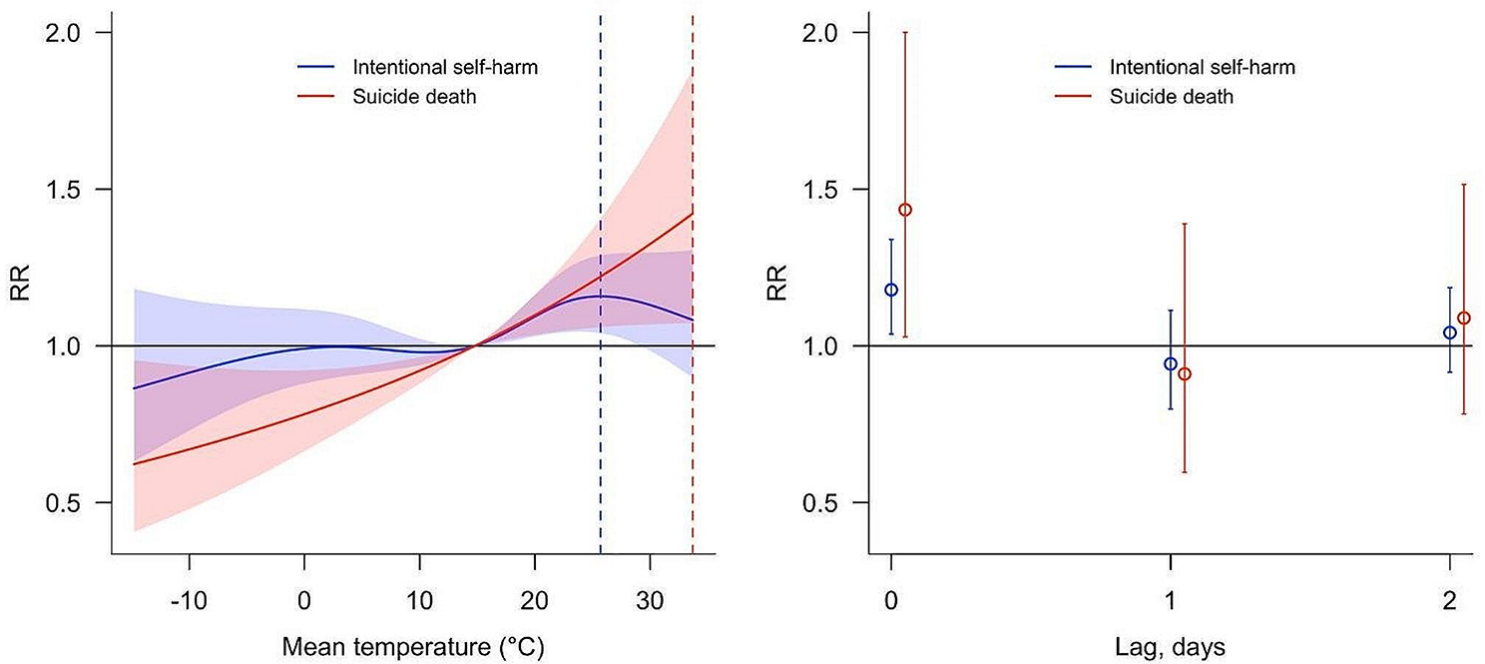
Cumulative association between mean temperature and suicides (Left) versus lag patterns of risks (Right). *Note: Dotted vertical lines indicate the maximum cumulative effect estimates for suicide deaths (in red) and ISH (in blue), while the shaded regions represent the 95% confidence intervals for each case

The left panel of Fig. 3 shows the overall influence of daily mean temperature on ISH and suicide deaths. For suicide deaths, the risk significantly increased with rising temperature, peaking at 33.7 °C relative to the 50th percentile of mean temperature. Conversely, the risk of ISH peaked at 25.7 °C. The cumulative RR for mean temperature over lag days was higher for suicide deaths (RR = 1.42, 95% CI: 1.07, 1.88) than for ISH (RR = 1.16, 95% CI: 1.04, 1.29). The right panel of Fig. 3 displays the lag-specific RR of daily mean temperature on ISH and suicide deaths. Significant associations were found exclusively at lag 0, where the RR for suicide deaths was 1.43 (95% CI: 1.03, 2.00), higher than the RR for ISH at 1.18 (95% CI: 1.04, 1.34).

### Subgroup analysis

**Fig. 4:**
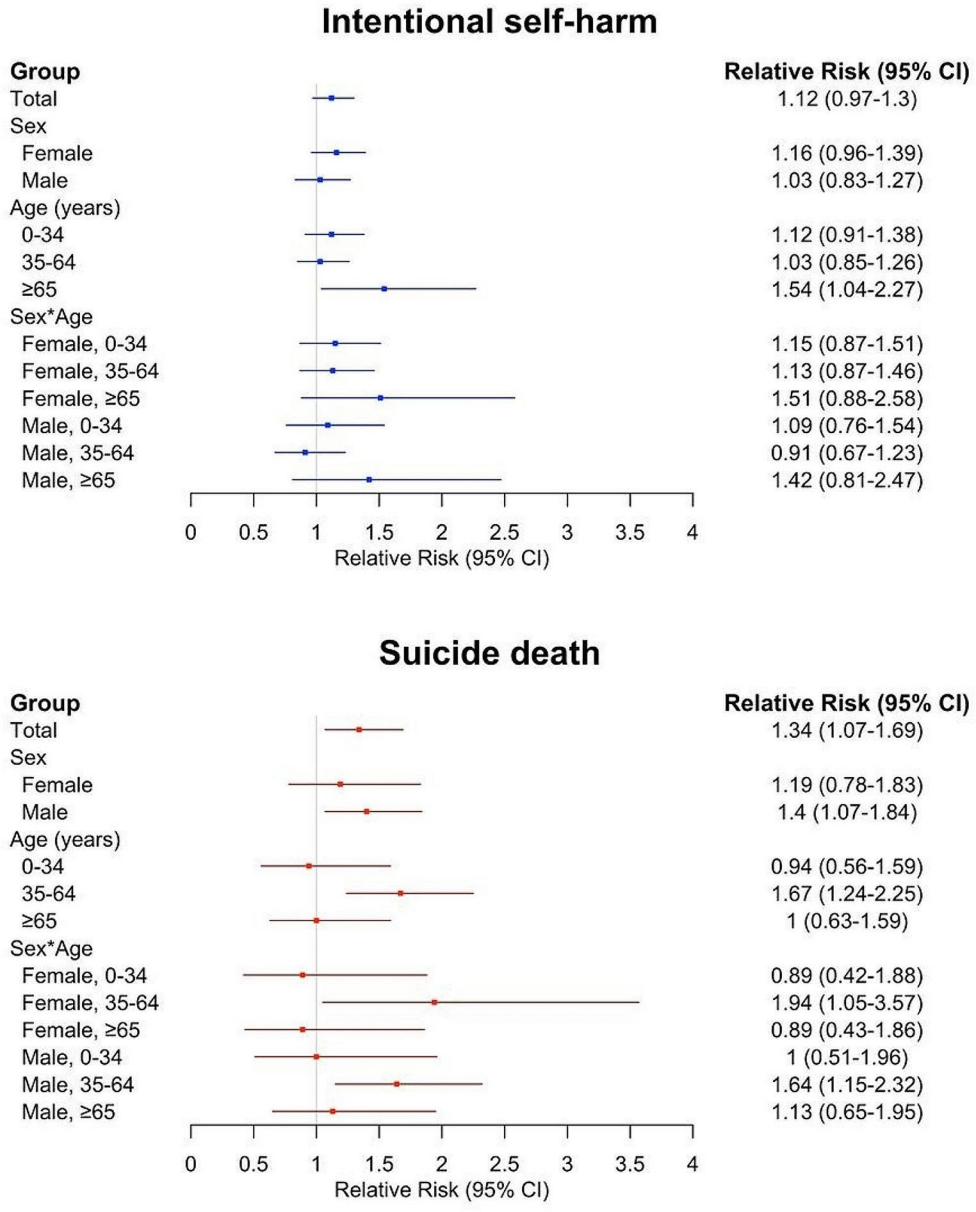
Cumulative relative risks of intentional self-harm and suicide deaths across different groups. *Note: Comparison is based on the 99th percentile of daily mean temperature versus the 50th percentile of daily mean temperature

Figure 4 shows the RRs at the 99th percentile of mean temperature compared to the 50th percentile reference temperature across various subgroups. In different subgroups, the association between temperature and ISH was borderline significant in females (RR = 1.16, 95% CI: 0.96, 1.39); but insignificant in males (RR = 1.03, 95% CI: 0.83, 1.27). Conversely, the association between temperature and suicide deaths was significant in men (RR = 1.40, 95% CI: 1.07, 1.84) but insignificant in women (RR = 1.19, 95% CI: 0.78, 1.83). The nonlinear association curves can be found in Supplementary Fig. S2. In the male group, a monotonic increase in the risk of ISH and suicide deaths was observed with rising temperatures. Conversely, the female group displayed an inverse J-shape, particularly pronounced in the ISH female group, where the risk peaked around 27 °C before declining.

In the age-specific subgroups, the association between temperature and ISH and suicide deaths varied. For individuals aged 65 years or older, the highest RR for ISH was observed (RR = 1.54, 95% CI: 1.04, 2.27). In contrast, the highest RR for suicide deaths was found in the 35–64 years group (RR = 1.67, 95% CI: 1.24, 2.25) (Fig. 4). Moreover, only the 35–64 years age group showed a monotonically increasing association between temperature and the risk of suicide death (Supplementary Fig. S3).

When evaluating sex and age subgroups, a significant association between temperature and suicide was identified exclusively in suicide deaths. No significant association was found in any ISH subgroup. For suicide deaths, the cumulative association was most pronounced for females aged 35–64 (RR = 1.94, 95% CI: 1.05, 3.57), followed by males aged 35–64 (RR = 1.64, 95% CI: 1.15, 2.32). The nonlinear association curves for all subgroups are available in Supplementary Fig. S4.

### Sensitivity analysis

In the sensitivity analysis, the DLNM findings showed considerable robustness when varying lag days and meteorological variables (Supplementary Table S2). Adjusting the lag days from 2 to 6 or using minimum or maximum temperatures instead of mean temperatures did not significantly alter the results.

## Discussion

This study seeks to distinguish between non-fatal suicide and fatal suicide deaths, examining their relationship with temperature. The analysis revealed varying association between temperature and ISH versus suicide deaths. Additionally, as temperature rises, the risk of both ISH and suicide deaths increases. Furthermore, at higher temperatures, the relative risk of severe outcomes, such as suicide deaths, becomes more pronounced compared to the risk of less severe ISH incidents. These findings clarify the distinct propensities for ISH and suicide death at elevated temperatures and suggests that evaluating them together may obscure their separate risk.

All suicide deaths are preceded by suicide attempt. However, Cause of Death database does not provide a breakdown of suicide attempts. To address this, an additional analysis of 36,936 cases from the NEDIS was conducted, including cases where the “emergency treatment results” outcome was death. Supplementary Fig. S5 shows that the findings were not significantly different from the ISH results that excluded fatal outcomes. The additional 1,418 deaths represented only 3.8% of the total, which had a minimal influence on the overall results. Supplementary Fig. S6, which display the cumulative relative risk of total ISH and suicide death across different groups, shows results consistent with those in Fig. 4.

The findings support the established association between high temperatures and mental health issues, including suicide (Frangione et al. 2022). They suggest that females and males aged 35–64 years are particularly sensitive to unusually high temperatures with regard to suicide risk. This indicates the presence of temperature-related risk factors that may contribute to variations in risk by age and sex. For example, temperature-related sleep disturbances and reduced parasympathetic activity could be mechanisms that precipitate suicide. Additionally, the increased likelihood of suicide in these subgroups at higher temperatures may be associated with factors such as acute alcohol poisoning or greater susceptibility to social isolation.

The complex interaction between sleep deprivation and parasympathetic withdrawal may directly or indirectly heighten the risk of suicide. Recent studies have shown that elevated nocturnal temperatures worsen sleep deprivation (Obradovich et al. 2017; Xu et al. 2021). This sleep deprivation, resulting from elevated temperatures, is believed to heighten the risk of ISH and suicidal death (Bernert et al. 2015). Additionally, high temperatures lead to more pronounced parasympathetic withdrawal (Abellán-Aynés et al. 2021). Research examining the relationship between parasympathetic withdrawal and both suicidal ideation and non-suicidal self-injury indicates that a decrease in respiratory sinus arrhythmia, a marker of the parasympathetic nervous system activity, is associated with an increased risk of suicidal ideation and may predict self-injury (Fox et al. 2018; Giletta et al. 2017). This connection between elevated temperature and parasympathetic withdrawal is further supported by studies showing that parasympathetic withdrawal occurs following sleep deprivation (Tobaldini et al. 2017).

To clarify the association between unusually high temperatures and suicide deaths particularly among males, initial attention was given to the prevalence of heavy drinking, a known risk factor for suicide. A study conducted in Korea found a positive correlation between heavy drinking and suicide rates (Kim 2020). This study focused on emergency patients with severe acute alcohol symptoms rather than the general population; however, evidence reveals that the risk of acute alcohol poisoning increases with temperature (Hensel et al. 2021). Additionally, a broader study conducted in Korea observed a 2% increase in beer sales for every one-degree rise in temperature (Kim 2012). Alcoholism is known to disrupt decision-making by causing dopaminergic dysregulation (Schindler et al. 2016), and acute alcohol poisoning is associated with fatal outcomes, including suicide, particularly among men (Kaplan et al. 2013). A follow-up study conducted in Korea, where firearm access is restricted, highlighted the distinct role of alcohol in ISH and suicide deaths. This study suggests that alcoholism may contribute to impulsive ISH (Kim et al. 2018).

Social isolation is a significant risk factor for suicide. A study in 2022 found a stronger correlation between social isolation and suicide in men than women, highlighting its role in explaining the higher suicide rates among men (Motillon-Toudic et al. 2022). Studies conducted in Korea show that reduced social interaction, especially among older men, can lead to adverse effects, including depression (Moon et al. 2010). In this demographic, social isolation may exacerbate difficulties in coping with elevated temperatures.

A recent systematic review and meta-analysis found a significantly stronger association between temperature and suicide mortality (RR = 1.052, 95% CI: 1.031, 1.072) than ISH (RR = 1.014, 95% CI: 1.003, 1.025) across various locations (Frangione et al. 2022). These findings align with our observation that temperature has a more pronounced effect on suicide deaths, which are severe outcomes. However, the RR for ISH in the meta-analysis did not distinctly separate suicide deaths from ISH, which slightly differs from our findings. Our study specifically examined completed ISHs resulted in death versus those that did not. Research that simultaneously evaluates suicide deaths and ISH within the same population is limited. A primary reference is a study conducted in California, USA, which compared suicide deaths to emergency room visits for mental illness. This study found that a 1 °F increase in mean temperature over 1 month corresponded to a 0.48% increase in emergency room visits for mental illness and a 0.81% increase in suicide deaths (Wang et al. 2014).

Subgroup analysis by sex and age revealed several significant findings. ISH is known to occur more frequently in females than in males (Kim et al. 2018). However, the association between high temperature and ISH seems comparable between both sexes, aligning with previous Japanese research that found no significant sex differences in self-harm (Kubo et al. 2021). Conversely, the association between high temperature and suicide deaths was more pronounced in males than in females. This finding aligns with the findings of a previous systematic review and meta-analysis, which shows a stronger association between high temperature and suicide among men (RR = 1.043, 95% CI: 0.996, 1.090) than women (RR = 1.007, 95% CI: 0.991, 1.024) (Frangione et al. 2022). Analysis by age groups revealed variation in ISH and suicide deaths; however, the association with high temperature was generally more significant among individuals aged 35–64 years. This strong association between mean temperature and suicide deaths in this age group supports the findings of a previous study conducted in Korea (Kalkstein et al. 2019).

Previous studies have not thoroughly investigated the interaction between sex and age, largely owing to limitations associated with small sample sizes. This issue is reflected in our findings, which revealed broad confidence intervals when analyzing female suicide deaths, also a result of small sample sizes. Figure 4 highlights the need for cautious interpretations of these results. Supplementary Fig. S4 and Supplementary Table S3 further illustrate that the MaxRT varies across different subgroups. Consequently, Fig. 4 shows that the RR of the 99th percentile temperature compared to the 50th percentile temperature may be underestimated in certain subgroups (such as females aged 65 years or older for suicide deaths, or males aged 65 years or older for ISH). This underestimation may lead to reduction in the observed RR at higher temperatures.

To the best of our knowledge, the present study offers several significant advantages. It expands on previous research conducted in Korea by examining the short-term association between temperature and suicides. The inclusion of ISH cases adds novel dimension, complementing the analysis of suicide deaths. Furthermore, efforts were made to enhance the accuracy of estimating the association between ambient temperature and suicide rates by categorizing subgroups by sex and age. This comprehensive approach helps elucidate the distinct influences of temperature on ISH and suicide deaths. This was achieved by using different settings for the nonlinear exposure-response and lag-response associations within the distributed lag nonlinear model. The robustness of the findings was confirmed through a sensitivity analysis, which involved varying the lag days and measuring different temperature types.

This study had some limitations. First, it was limited to Seoul, Korea. making it difficult to generalize the findings to other regions with different temperature distributions and demographics. Second, ambient temperature, the primary exposure, was estimated from a single fixed monitoring site, potentially introducing errors in exposure measurement. Third, the model did not account for air pollution, a well-established confounding factor positively associated with ISH and suicide deaths (Braithwaite et al. 2019; Davoudi et al. 2021). Fourth, socioeconomic variables that influence ISH and suicide deaths were not considered. For instance, relative income deprivation has been positively correlated with suicidal ideation in Korea (Pak and Choung 2020). Additionally, owing to the limited sample size, the results of the subgroup analysis may lack consistency. Future studies should account for air pollution as a time-varying potential confounder and explore socioeconomic status as a potential effect modifier.

## Conclusions

The study investigated the relationship between average temperatures and ISH or suicide deaths in Seoul, Korea, from 2014 to 2019. The DLNM was used to estimate the nonlinear and delayed effects of temperature more accurately. The findings revealed that unusually higher temperatures significantly increased the risk of ISH and suicide deaths, with males being more vulnerable to rising temperatures than females. Additionally, middle-aged (35–64) male and female were found to be the most vulnerable to the effects of high temperatures.

The findings indicate that short-term exposure to high temperatures can trigger both ISH and suicide deaths, with a stronger association observed for suicide deaths. These insights are vital for creating adaptive responses to climate change, particularly concerning how elevated temperatures affect ISH and suicide rates. Strategies should not only focus on reducing mortality but also address overall morbidity. Additionally, it is crucial to assess temperature-related risks for different sex and age subgroups separately. These findings offer important guidelines for creating more personalized suicide prevention policies.

## Electronic supplementary material

Below is the link to the electronic supplementary material.

Supplementary Material 1
