# Supplementary material for: Association of ambient temperature with intentional self-harm and suicide death in Seoul: a case-crossover design with a distributed lag nonlinear model
Source: Int J Biometeorol. 2024 Aug 23;68(11):2321–31. doi: 10.1007/s00484-024-02752-z (PMC11519195; doi:10.1007/s00484-024-02752-z)
Supplement: Supplementary file 1 — Supplementary Material 1 [file 484_2024_2752_MOESM1_ESM.docx]

**Supplementary Material for**

**Association of ambient temperature with intentional self-harm and suicide death in Seoul: A case-crossover design with a distributed lag non-linear model**

Seunghyeon Kim^1^, Yoonhee Kim^2^ and Eunsik Park^1*^

^1^ Department of Mathematics and Statistics, Chonnam National University, 77 Yongbong-ro, Buk-gu, Gwangju, 61186. Korea.

^2^ Department of Global Environmental Health, Graduate School of Medicine, The University of Tokyo, Tokyo 113-0033, Japan.

^*^Corresponding author

Eunsik Park

Email: [espark02@jnu.ac.kr](mailto:espark02@jnu.ac.kr)

Contents

Supplementary Table S1

Supplementary Table S2

Supplementary Fig. S1

Supplementary Fig. S2

Supplementary Fig. S3

Supplementary Fig. S4

Supplementary Fig. S5

Supplementary Fig. S6

Supplementary Table S3

Additional File 1

**Supplementary Table S1** Quasi-Akaike information criterion values for the association between mean temperature and intentional self-harm, suicide death according to various distributed lag non-linear model specifications

|  | Intentional self-harm | Suicide death |
| --- | --- | --- |
| 1 knot at 50th percentile | 12011.10 | 9689.27* |
| 2 knots at |  |  |
| 3.3rd and 66.7th | 12015.85 | 9694.17 |
| 25th and 75th | 12016.33 | 9694.44 |
| 10th and 90th | 12016.59 | 9694.67 |
| 3 knots at |  |  |
| 25th, 50th, and 75th | 12008.38* | 9699.49 |
| 10th, 75th, and 90th | 12010.51 | 9698.77 |
| 10th, 50th, and 90th | 12009.42 | 9698.96 |

*Main model

**Supplementary Table S2** Sensitivity analyses by changing lag days and temperature variables

| Models | Intentional self-harm | Suicide death |
| --- | --- | --- |
| Lag period, days |  |  |
| 2 | 1.16 (1.04, 1.29) | 1.42 (1.07, 1.88) |
| 3 | 1.17 (1.05, 1.31) | 1.39 (1.03, 1.86) |
| 6 | 1.22 (1.07, 1.38) | 1.46 (1.05, 2.03) |
| Temperature |  |  |
| Minimum temperature | 1.13 (0.91, 1.40) | 1.45 (1.02, 2.06) |
| Mean temperature | 1.16 (1.04, 1.29) | 1.42 (1.07, 1.88) |
| Maximum temperature | 1.11 (1.01, 1.21) | 1.30 (1.03, 1.62) |


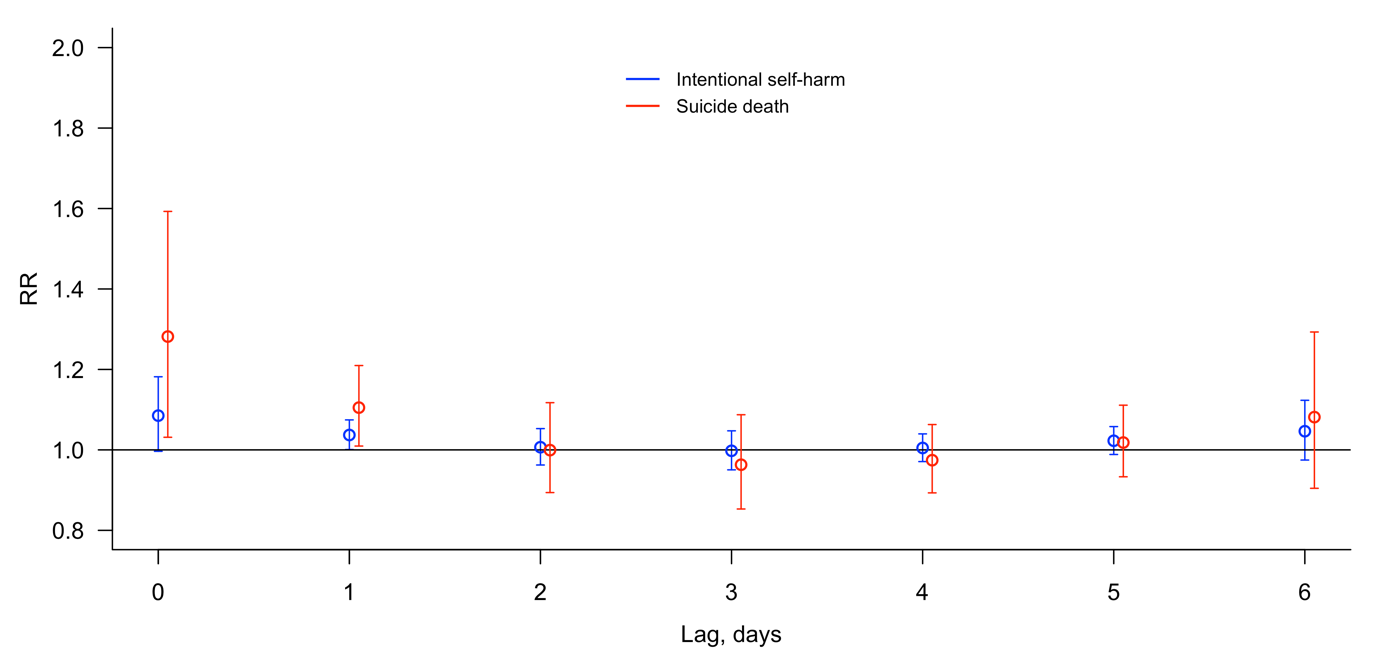


**Supplementary Fig. S1** The lag pattern of risks of intentional self-harm and suicide death at the maximum risk temperature from the same day (lag 0) to 6 days prior to the given day (lag 6)


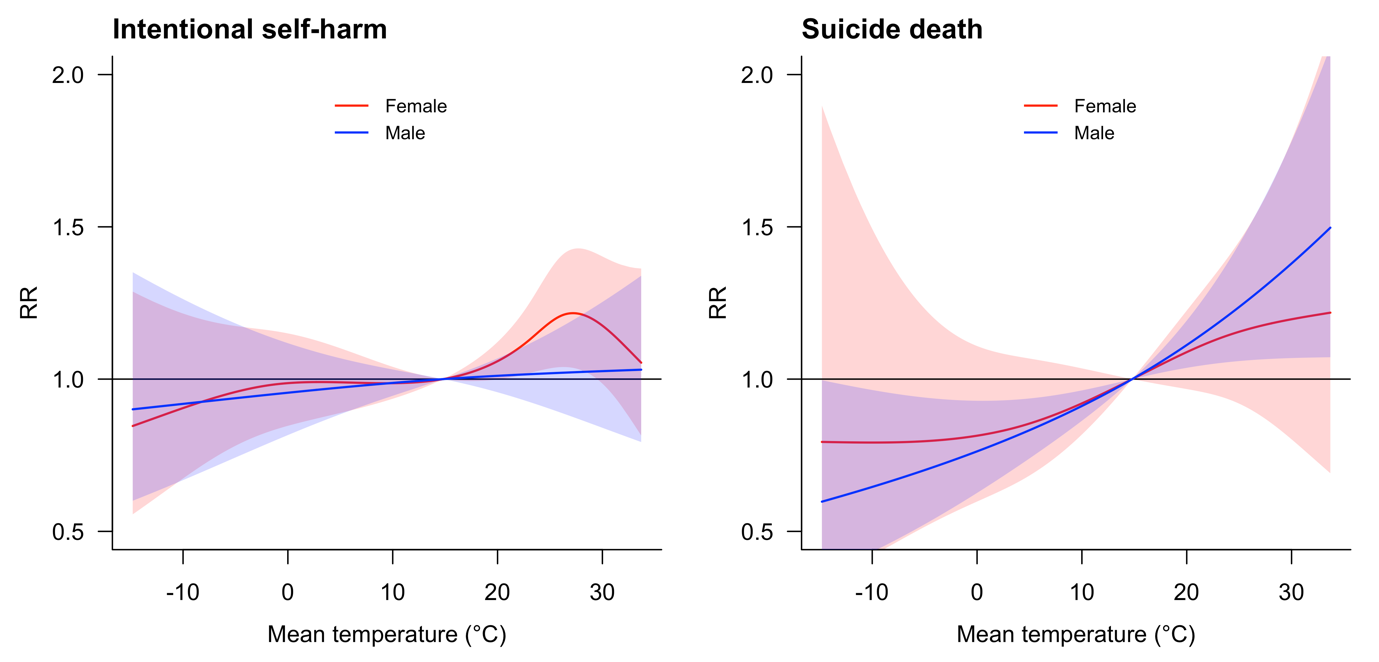


**Supplementary Fig. S2** Overall cumulative association of mean temperature with intentional self-harm and suicide death across lag 0-2 days by sex


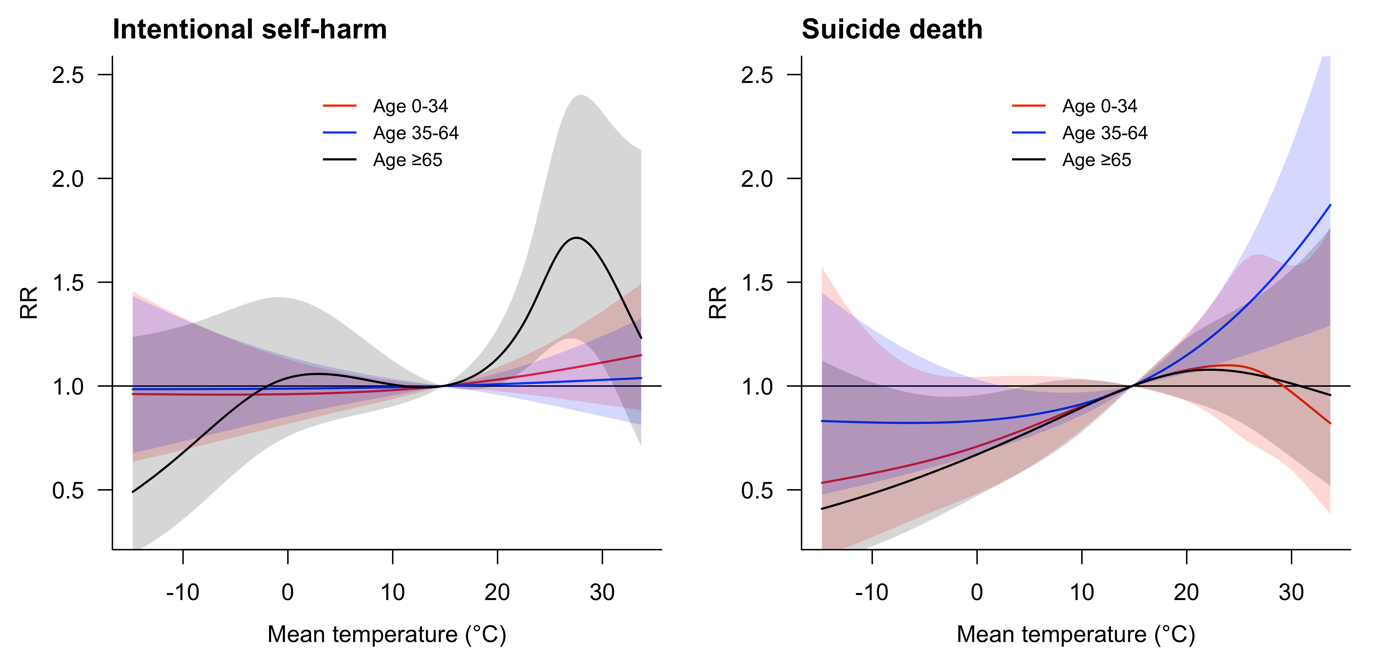


**Supplementary Fig. S3** Overall cumulative association of mean temperature with intentional self-harm and suicide death across lag 0-2 days by age


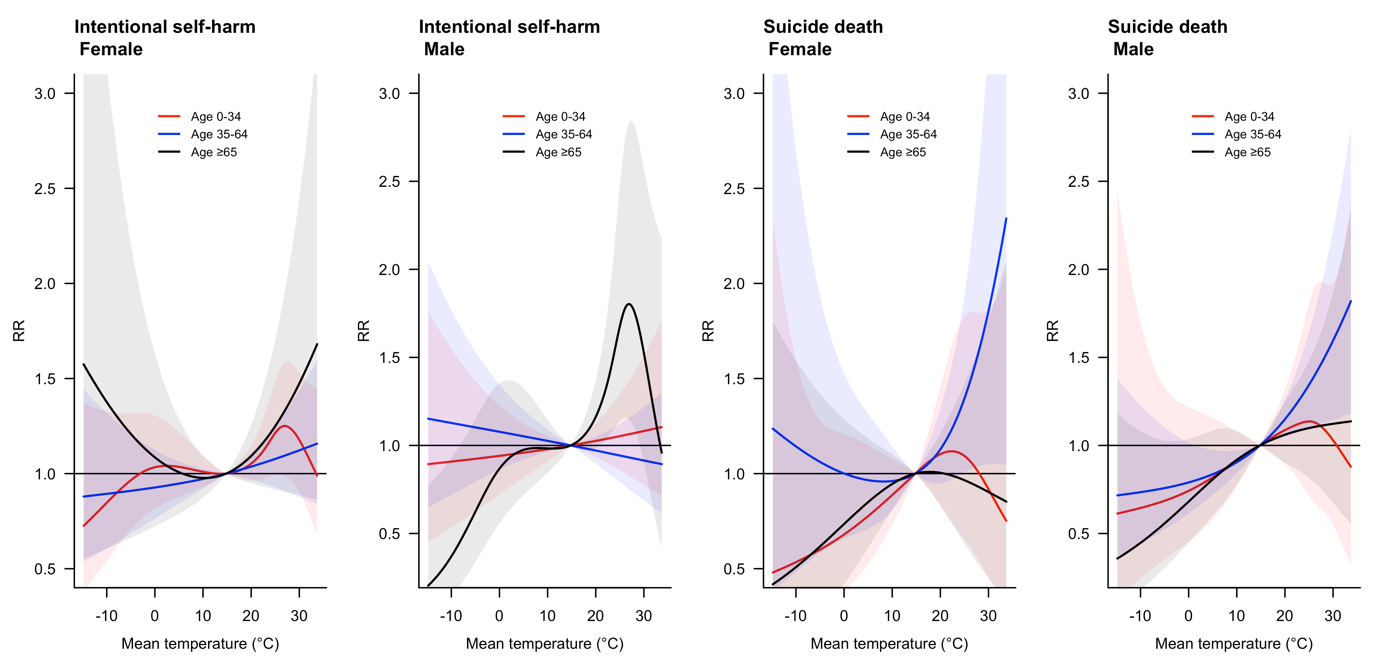


**Supplementary Fig. S4** Overall cumulative association of mean temperature with intentional self-harm and suicide death by sex and age

**
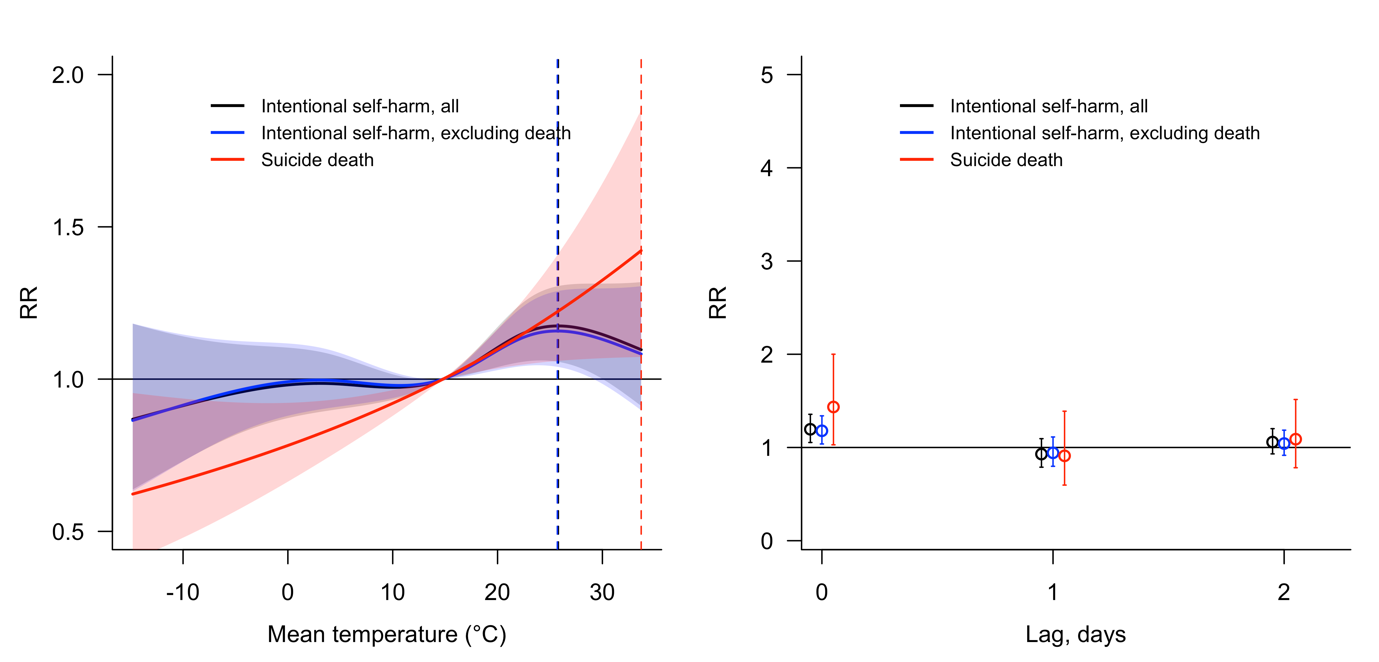
 Supplementary Fig. S5** Cumulative association between mean temperature and suicides (Left) versus lag patterns of risks (Right).


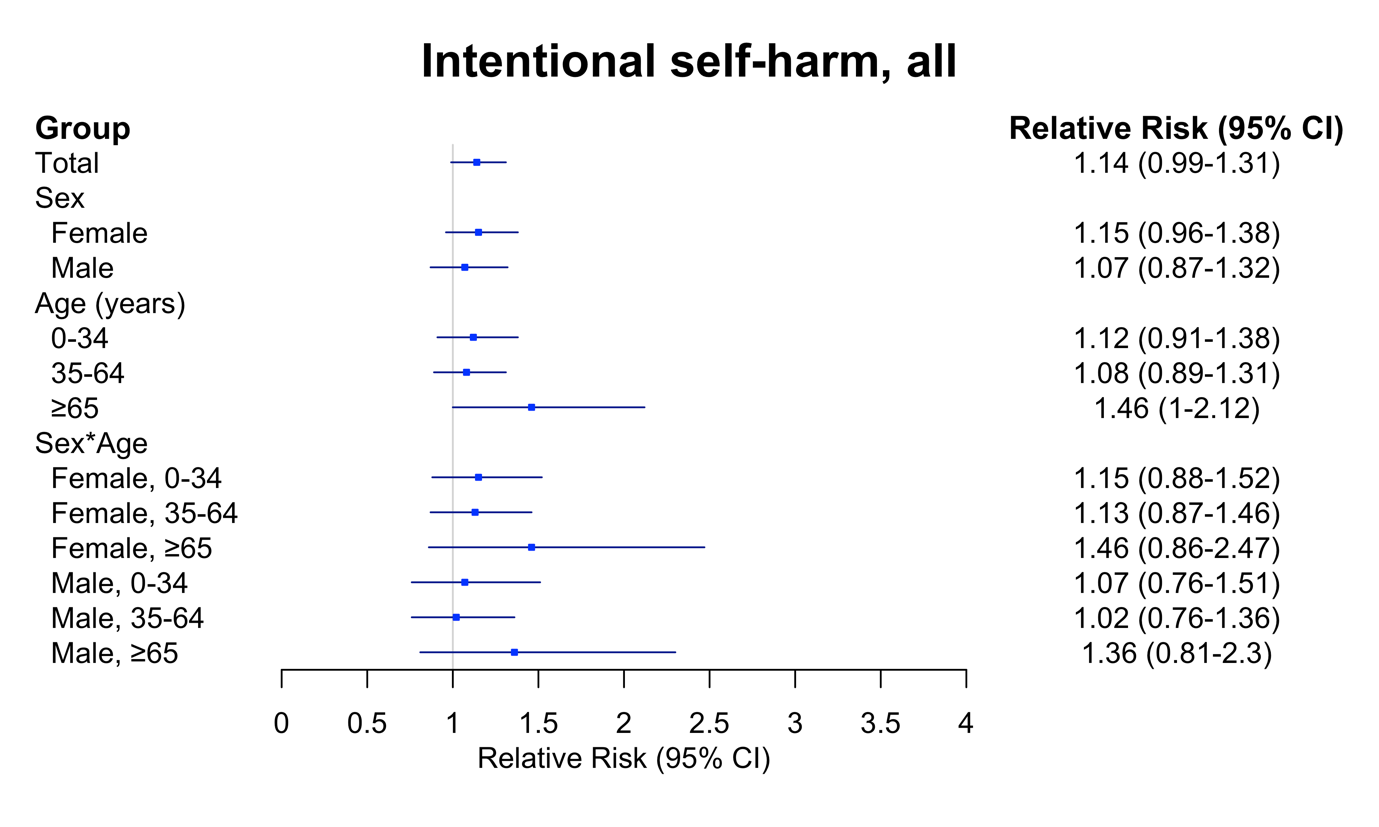


**Supplementary Fig. S6** Cumulative relative risks of all intentional self-harm cases across different groups.

| **Supplementary Table S3** The relative risk of intentional self-harm and suicide death and maximum risk temperature (°C) | | | | | |
| --- | --- | --- | --- | --- | --- |
|  | Intentional self-harm |  |  | Suicide death |  |
|  | MaxRT (°C) | RR |  | MaxRT (°C) | RR |
| Total | 25.7 | 1.16 (1.04, 1.29) |  | 33.7 | 1.42 (1.07, 1.88) |
| Sex |  |  |  |  |  |
| Female | 27.2 | 1.22 (1.04, 1.43) |  | 33.7 | 1.22 (0.69, 2.15) |
| Male | 33.7 | 1.03 (0.79, 1.34) |  | 33.7 | 1.50 (1.07, 2.09) |
| Age (years) |  |  |  |  |  |
| 0-34 | 33.7 | 1.15 (0.88, 1.49) |  | 23.6 | 1.04 (0.82, 1.32) |
| 35-64 | 33.7 | 1.04 (0.81, 1.33) |  | 33.7 | 1.96 (1.29, 2.98) |
| ≥ 65 | 27.5 | 1.71 (1.23, 2.40) |  | 24.9 | 1.06 (0.82, 1.37) |
| Sex*Age |  |  |  |  |  |
| Female, 0-34 | 27.0 | 1.25 (0.99, 1.58) |  | 18.1 | 1.01 (0.91, 1.13) |
| Female, 35-64 | 33.7 | 1.16 (0.84, 1.60) |  | 33.7 | 2.50 (1.10, 5.71) |
| Female, ≥ 65 | 33.7 | 1.68 (0.86,3.27) |  | 20.6 | 1.10 (0.78, 1.55) |
| Male, 0-34 | 33.7 | 1.10 (0.71, 1.71) |  | 22.8 | 1.10 (0.83, 1.45) |
| Male, 35-64 | -14.8 | 1.15 (0.65, 2.04) |  | 33.7 | 1.82 (1.18, 2.81) |
| Male, ≥ 65 | 26.9 | 1.80 (1.15, 2.83) |  | 33.7 | 1.13 (0.59, 2.15) |
| MaxRT, Maximum risk temperature; RR, Relative risk  *Note: MaxRT was defined between the 1^st^ and 99^th^ percentiles of the mean temperature distribution as the highest risk of intentional self-harm and suicide death | | | | | |
